# BPI-ANCA and Long-Term Prognosis among 46 Adult CF Patients: A Prospective 10-Year Follow-Up Study

**DOI:** 10.1155/2012/370107

**Published:** 2012-12-30

**Authors:** Ulrika Lindberg, Malin Carlsson, Claes-Göran Löfdahl, Mårten Segelmark

**Affiliations:** ^1^Department of Respiratory Medicine and Allergology, Lund University and Skane University Hospital, 221 85 Lund, Sweden; ^2^Department of Nephrology and Department of Clinical Sciences, Lund University, 221 85 Lund, Sweden; ^3^Department of Medicine and Health, Linkoping University, 590 50 linkoping, Sweden; ^4^Department of Nephrology UHL, County Council of Ostergotland, 581 85 Linkoping, Sweden

## Abstract

*Introduction*. Anti-neutrophil cytoplasmic antibodies specific for bactericidal/permeability-increasing protein (BPI-ANCA) are frequent in CF patients and mainly develop in response to infection with *Pseudomonas aeruginosa*. It is not known to what extent BPI-ANCA correlates to prognosis. *Objectives*. To evaluate the prognostic value of IgA-BPI-ANCA, measured at the beginning of the study, for transplantation-free survival. *Methods*. A cohort of 46 adult, nontransplanted CF patients was generated, 1995–1998, and characterized using Leeds criteria, lung function, and IgA-BPI-ANCA levels measured by ELISA. The cohort was followed until December 2009, using the combined endpoint of death or lung transplantation. *Results*. Lung function and IgA-BPI-ANCA, but not Leeds criteria, were significantly associated with adverse outcome. No patient with normal lung function at baseline reached endpoint. Within 10 years 8/11 with high BPI-ANCA reached an endpoint compared to 3/17 ANCA-negative patients. A similar result was seen within the Leeds I group where 7 out of 9 BPI-ANCA-positive patients reached endpoint, compared to none of the 5 patients without BPI-ANCA. *Conclusions*. IgA-BPI-ANCA is associated with adverse outcome among *Pseudomonas aeruginosa* infected CF patients, suggesting that BPI-ANCA is a biomarker of an unfavourable host-pathogen interaction.

## 1. Introduction

Cystic fibrosis (CF) is a disease with multiple clinical manifestations, where the prognosis for the individual often is difficult to foresee. The lack of reliable predictors of the disease course is a well-recognized problem in CF patients [1]. Pulmonary disease is the main determinant of morbidity and mortality in CF [2] and hence it is important to identify factors that can explain and predict variations in lung function.

The genetic correlation between pancreatic sufficiency and milder disease has been established since it became possible to genotype CF patients [3]. In a longitudinal study including the whole Swedish CF population it was shown that CFTR genotypes found in conjunction with long-term pancreatic sufficiency phenotype were associated with a better pulmonary function [1].

ΔF508 is the most common mutation, with an allele frequency of 70% in Swedish CF patients. Homozygote patients generally have a more severe clinical phenotype than ΔF508 heterozygotes and patients with no F508 allele, although substantial phenotypic variability is seen [2, 4]. McKone et al. [2] studied CFTR genotype as a predictor of prognosis in CF and found that patients with a high risk CFTR genotype had a greater than two fold risk of death compared to patients with a low risk genotype.

*Pseudomonas aeruginosa* (*PsA*) colonization is a well-established risk factor in CF. In patients who subsequently died or became subjects for lung transplantation, chronic *PsA* colonization was significantly more frequent [5]. FEV1 is the variable of lung function that best reflects the progression of lung disease in CF; impaired vital capacity (VC) is seen only in late stages of the disease [6].

Bactericidal/permeability-increasing (BPI) protein is a protein found in the azurophilic granules of neutrophil granulocytes. BPI has a potent antimicrobial activity against Gram-negative bacteria, such as *PsA*, by neutralising the endotoxin and by playing a part in opsonization of the bacteria. [7–13]. Anti-neutrophil cytoplasmic antibodies (ANCA) with BPI specificity have been identified in different diseases associated with Gram-negative bacteria, such as inflammatory bowel diseases (IBD) and primary sclerosing cholangitis [14], and are frequently present in CF patients [11, 15, 16]. 

BPI-ANCA seems to develop in response to *PsA* colonization, but there are also patients colonized with *PsA* who do not develop BPI-ANCA [17]. After eradication of *PsA *colonization by lung transplantation a significant decrease in BPI-ANCA levels has been seen [17] and in a recently published study [18] it was shown that BPI-ANCA levels significantly decreased after sinus surgery. Exactly why BPI-ANCA is produced is a so far poorly understood process. One theory is that chronic infections stimulate extensive release of BPI triggering autoantibody production [16]. Schultz suggested that chronic Gram-negative infection accompanied by local neutrophil accumulation results in the delivery of BPI-coated particles to immature dendritic cells thereby inducing autoimmunity to BPI [19].

Earlier publications from our study group have shown a correlation between the presence of BPI-ANCA and reduced lung function in CF [17, 20, 21]. A high level of BPI-ANCA was associated with more severe lung disease both when measured with radiology and spirometry [16, 20, 22]. The presence of BPI-ANCA has also been associated with a higher number of antibiotic courses, low body mass index, the presence of resistant *PsA*, CF related liver disease, hypergammaglobulinemia, male sex, and inflammatory syndrome [23]. In a previous study we also found indications that the presence of BPI-ANCA predicted outcome [17].

We have now extended this followup to more than 10 years for all patients. The aim of this prospective study was to follow the progress of lung disease in 46 adult CF patients to elucidate the significance of a positive IgA-BPI-ANCA as a prognostic factor, in relationship to lung function and pseudomonas colonization status. 

## 2. Patients and Methods

A cohort was generated in 1995–1998, as described earlier by Carlsson et al. [21]. Forty-six patients, all regular adult nontransplanted patients at the Lund CF Center, were included in the cohort. All patients at the centre were eligible for the study and 46 out of the 54 patients who were registered during the inclusion period were included. Reasons for not participating were trivial, such as not seeing a nurse assigned to the study during the inclusion period. The mean age of the patients at inclusion was 24.6 years (range 18.4–44.6); twenty were female, twenty-six male (Table 1). The study was approved by the Ethical Committee at Lund University and all participants gave their written informed consent. 

The CF diagnosis was confirmed genetically in all cases as part of the clinical routine and the results of mutation analyses and all other clinical data were obtained from patient records. When the patients were subdivided into groups according to CFTR genotype (A, homozygosity for ΔF508; B, severe/severe mutation, i.e., one class I, class II, or class III mutation on each allele including heterozygosity for ΔF508; C, one or two missense mutations, i.e., class IV or V mutations; D, one or two unknown mutations), no significant differences in lung function, bacterial colonization, or BPI-ANCA levels were seen between the genotype groups at inclusion [24].

The patients were followed prospectively until December 31, 2009. Endpoint was death or lung transplantation, treated as equal.

### 2.1. Statistical Analysis

Statistical calculations were performed using SPSS for Windows version 19. Survival curves were estimated using Kaplan-Meyer method. Log rank tests were used to compare survival between subgroups. Cox proportional hazard regression was used to estimate hazard ratios. 

### 2.2. Lung Function

FEV1 was measured by spirometry at the Department of Clinical Physiology, Lund University Hospital, on a yearly basis, following the guidelines from the American Thoracic Society [25]. The results were expressed as proportion of predicted values (FEV1% pred) calculated according to Quanjer et al. [26] from the patients' height, age, and sex. The lung function was categorized into three groups based on the spirometry results: normal lung function, FEV1 >80% pred; moderate lung damage, FEV1 50–80% pred; and severe lung damage, FEV1 <50% pred.

### 2.3. BPI-ANCA

IgA BPI-ANCA was analyzed by ELISA and measured at the time of inclusion. IgA-BPI-ANCA was measured again after 5 and 10 years in those patients who until that date had not reached an endpoint. Purified BPI was obtained from Wieslab AB (Lund, Sweden) and direct binding was performed [27]. In short, antigens were coated onto microtiter plates at a concentration of 1 microliter/mL in a bicarbonate buffer. Serum samples were diluted 1/80 and incubated for one hour. Bound antibodies were detected using alkaline phosphatase-conjugated goat anti-human IgA (Eurodiagnostica, Malmö, Sweden). IgA-BPI-ANCA was quantified from a calibrator curve that was serially diluted and the results expressed as arbitrary units (U). The cut-off level for IgA BPI-ANCA was determined to be > or = 67 arbitrary units (U/L) from the mean absorbance value of 42 normal paediatric sera + 3SD [17] and levels over 200 (three times the positive level) were arbitrarily considered as high. The level 200 was chosen independently of results and was created to separate moderate BPI-ANCA levels from high levels.

### 2.4. Bacterial Colonization

Samples for respiratory secretion cultures were taken when the patient attended a routine outpatient visit. Sampling, transport, and culture were performed according to routine procedures. History of bacterial colonization was obtained from patient records as far back as possible, and colonization was defined according to the Leeds criteria [28].

## 3. Results

The cohort of 46 adult CF patients was followed prospectively from inclusion 1995–1998 up until December 31, 2009. Death and lung transplantation were defined as endpoints. In general, the entire Swedish CF population is doing better for each decade, and life expectancy for CF patients, as a group, increases over time. Consistent with this notion the present cohort had, as a group, a fairly well preserved lung function, with a mean FEV1% pred of 70% at inclusion. Only thirteen of the patients had, at the beginning of the study, a lung function of less than 50% FEV1% pred. Twenty-six of the patients were chronically colonized with *PsA* at the start of the study. Even though the follow-up time in this study was over ten years, in some cases even as long as 14 years, 27 patients were still alive and not transplanted, at the final followup (59%).

In total seven patients reached an endpoint within five years after inclusion and 15 within ten years (Table 2). The ten year result includes three patients who died and 12 patients who received a lung transplant. One of the patients who died acquired colon cancer when he had a relatively good lung function. He was included in the study in 1995 (FEV1 78% pred), but in connection to his operation and treatment for colon cancer, he became *PsA* colonized and his lung function deteriorated rapidly. There is no doubt that his CF lung disease contributed to his death, but the main cause of death was the colon cancer. 

In the cohort there was only one patient colonized with *Burkholderia cepacia* at inclusion, and she was known to carry the bacteria from 1995. This patient had moderate lung damage for a very long time (FEV1 55% pred), which after pregnancy worsened and she was lung transplanted shortly after. None of the patients in the study was carrying MRSA.

In the cohort there were 26 male and 20 female patients. After five years three male and four female patients had reached an endpoint. After ten years five males and ten females were either dead or had received a lung transplant, and at the final followup nine males and ten females had experienced an endpoint. 

Eleven patients had insulin treated diabetes mellitus when the study began. 34 were not diabetic and for one patient information about diabetes was not available. At the time of final followup five of the diabetic patients (45,4%) and fourteen of the nondiabetic patients (41,2%) had reached endpoint.

### 3.1. Leeds Classification and Long-Term Outcome

**Figure 1:**
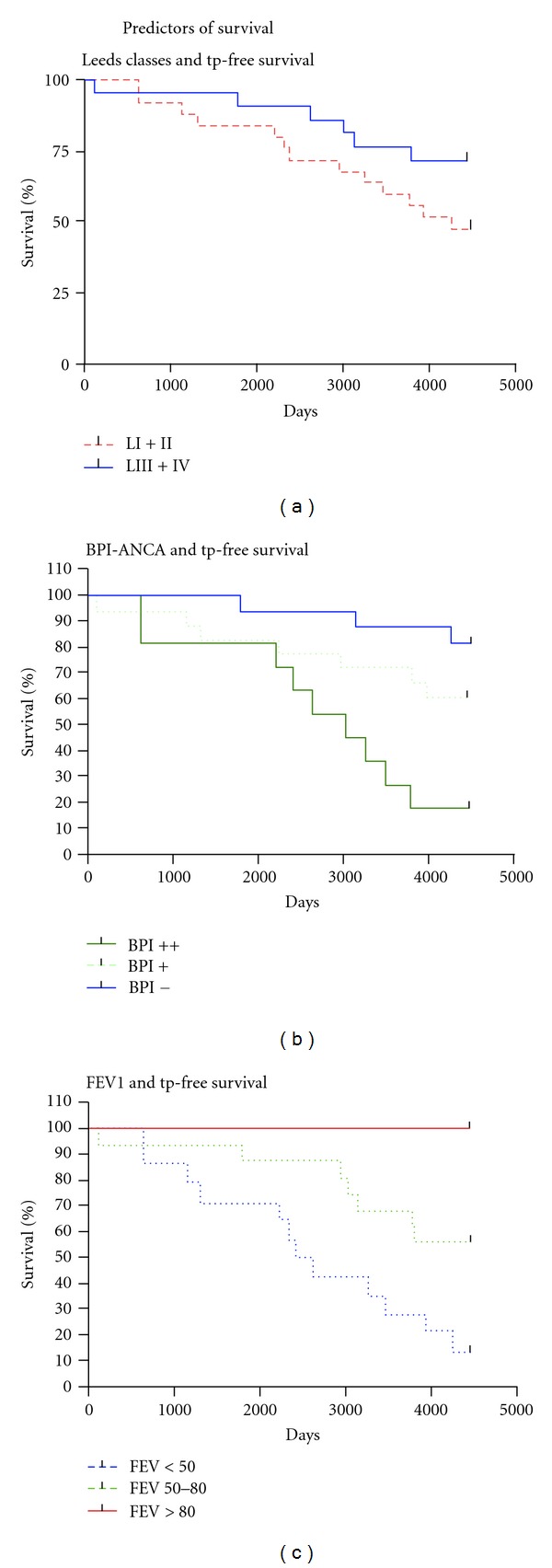
(a) PsA colonization and transplantation free survival. The blue line shows patients who are not chronically colonized with *PsA* (never had *PsA* or free from earlier colonization, Leeds class IV or III). The red line shows patients who are chronically or intermittently colonized with *PsA* (Leeds class I or II) (log rank test *P* = 0.13). (b) BPI-ANCA and days of transplantation free survival. The blue line indicates patients without IgA-BPI-ANCA, the dotted line patients with IgA-BPI-ANCA level of 67–200, and the green line patients with a BPI-ANCA above 200 at inclusion. There is a clear difference in transplantation free survival for BPI-negative patients compared to patients with a high BPI-ANCA (log rank test *P* = 0.002). (c) Lung function and transplantation free survival. Patients with FEV1 >80% pred, FEV1 between 50 and 80% pred, and FEV1 below 50% pred are indicated by red line, green dotted line, and blue dotted, respectively. None of the patients with a normal lung function at inclusion die or receive a lung transplant during over ten years of followup. The patients who have a low lung function at the start of the study experience a negative outcome to a very high degree (log rank test *P* < 0.001).

The well-known association between *PsA* colonization in CF patients and adverse clinical outcome can be seen also in this study (Table 2 and Figure 1(a)), but bacterial colonisation categorized by the Leeds classification was statistically not a significant determinant of outcome (*P* = 0.113). After ten years eleven (42%) out of the 26 patients belonging to Leeds I or II had experienced an endpoint, and at December 31, 2009, 54% were either dead or had received a lung transplant. Compared to this the patients who were free of earlier *PsA* (Leeds III) or who had never been infected with *PsA* (Leeds IV) did better. At the time of final follow-up only five of these patients (20%) had reached endpoint. The group of five patients includes the man with colon cancer and the woman with *Burkholderia cepacia*. These two patients are being marked by ∗ and ∗∗, respectively, in all tables. 

### 3.2. BPI-ANCA Level Is More Informative Than Leeds Classification

The association between IgA-BPI-ANCA level at inclusion and an adverse outcome is evident from Table 2. After ten years 15 patients had reached an endpoint; out of these only two (13%) were IgA-BPI-ANCA negative at inclusion. The median IgA-BPI-ANCA level of all patients reaching an endpoint within ten years was 251 ELISA units as compared to 69 for the 31 patients who did not experience such an event.

In total, 16 out of the 29 (55%) BPI-positive patients were either dead (*n* = 4) or had been lung transplanted (*n* = 12) on the date for the final followup. Out of the 17 IgA-BPI-negative patients only one patient died and two received a lung transplant; one of them is the woman with *Burkholderia cepacia.*

In contrast to Leeds groups, IgA-BPI-ANCA level was significantly correlated to outcome. The hazard ratio for one standard deviation of BPI-ANCA, used as a continuous variable, was calculated to 1.76 (95%; CI: 1.25–2.48; *P* ≤ 0.001). Also when comparing survival between subgroups based on IgA-BPI-ANCA as depicted in Figure 1(b) a significant result was obtained (log rank test, *P* = 0.002). Of particular interest is the finding that also when the analysis is confined to chronically colonized patients (Leeds I group), there is a significant hazard ratio for one standard deviation of IgA-BPI-ANCA (1.69; CI: 1.13–2.53; *P* = 0.011).

Chronic colonization with *PsA* is known to eventually lead to severe lung damage, as shown in earlier studies. Five patients in this cohort were colonized with *PsA* but had not developed IgA-BPI-ANCA at the time of inclusion (Table 2). None of these five patients reached endpoint within ten years, but one did before December 31, 2009. Their mean FEV1 at inclusion was 95% of predicted and after ten years their lung function was still surprisingly well preserved, mean FEV1 79% pred, considering that they are chronically colonized with *PsA,* a greater degree of deterioration in lung function would have been expected. After ten years of followup four of the patients still had not developed IgA-BPI-ANCA (one unknown), although they were all still *PsA* colonized. Four of these patients were pancreatic insufficient, one sufficient. As a comparison the mean FEV1 at inclusion among the chronically *PsA* colonized BPI-ANCA-positive patients was only 50% of predicted. 

BPI-ANCA is associated with *PsA* colonization and in this cohort, out of the 26 patients who are categorized as belonging to Leeds I and II, 21 were IgA-BPI-ANCA positive. However, we also found six patients who were IgA-BPI-ANCA positive belonging to Leeds group IV which means that they had developed BPI-ANCA without being known to have harboured *PsA* (Table 2). One was the patient with colon cancer mentioned above. When studying the course of the remaining five we found that all remained in Leeds class IV after five years of followup, indicating that IgA-BPI-ANCA did not predict colonization in these cases. However, ANCA levels fell in all these individuals and were negative in three after 5 years. The patient in this group with the highest titre at inclusion (244 units) did actually acquire a *PsA* infection between 5 and 10 years of followup. Overall these five patients followed a favourable course; only one developed severe pulmonary insufficiency and received a lung-transplant 10.5 years after inclusion in the study. 

### 3.3. Lung Function and Long-Term Outcome

Lung function at inclusion was a very important predictor for the long-term prognosis. As shown in Figure 1(c), none of the patients with a normal FEV1 at inclusion reached endpoint during the followup. Patients with a severe lung damage at inclusion reached endpoint to a very high degree, 11 out of 13 patients (Table 3). The hazard ratio for an increase in FEV1% pred with one standard deviation was 0.334 (0.18–0.60; *P* ≤ 0.001).

A positive IgA-BPI-ANCA was associated with low lung function at inclusion (Table 3). Mean FEV1 in the whole BPI-ANCA+ group was 58% pred (median 60%) and in the IgA-BPI-ANCA-negative group 87% pred (median 93%). The moderate sample size and the association between low lung function and adverse outcome in this cohort make it difficult to analyze whether IgA-BPI-ANCA provides any additional information when FEV% pred is known. But it is of interest to note, however, that among patients with severe lung damage all patients with high ANCA levels reached end point within ten year as compared to 3 out 7 with lower values (Table 3). When looking at patients with moderate lung function impairment, disregarding the patients reaching endpoint from non-*PsA* associated causes, two out of five patients with high ANCA reached an endpoint within ten years compared to two out of 10 patients with lower ANCA levels.

## 4. Discussion

In a disease as complex as CF tools to predict the prognosis would be very useful for the clinician, trying to find out which patient who best needs the most active therapy. In a world where the bacteria are getting more and more resistant and effective antibiotics are few it is important to use the pharmacological options we have at hand as effectively as possible and in an environmentally correct way. 

This study shows that presence of autoantibodies to the neutrophil granule constituent bactericidal/permeability-increasing protein (BPI-ANCA) of IgA class is strongly associated with an adverse outcome. End-stage lung disease, as indicated by death or lung transplantation, had after 10 years developed in 8 of 11 patients with high IgA-BPI-ANCA levels as compared to 2 of out 17 BPI-ANCA-negative patients. Overall there is a very strong correlation between levels of IgG and IgA-ANCA, even though some patients exhibit divergent results. For the sake simplicity we present in this study data only on IgA-BPI-ANCA, because our earlier studies have suggested that IgA-BPI-ANCA show a slightly better correlation with decreased lung-function. 

Leeds classification is an established tool to categorize *PsA* colonisation and thus to predict outcome [28]. When comparing the predictive capacity of Leeds classification with BPI-ANCA we found that a BPI-ANCA test taken at a single occasion was significantly correlated to outcome while the classification based on series of sputum cultivations was not. The main reason for this difference was the fact that Leeds I patients with negative ANCA tests had a very good prognosis. A probable explanation for this is that presence of ANCA heralds that an unfavourable host-pathogen interaction has taken place. 

Even though *Pseudomonas aeruginosa* infection is believed to be one main culprit for the progression of CF lung disease [29] a multitude of other factors influence the course. The complexity of the disease and the variability in phenotype make it difficult to foresee which patient is going to deteriorate in lung function. The *Burkholderia cepacia* complex is a well-known aggressive pathogen that can cause very fast deterioration in CF patients [30] and there are other pathogens where we are unsure about their capability of causing lung damage, for instance *Stenotrophomonas maltophilia* and different fungal infections. Complications to CF as diabetes mellitus and malnutrition play important roles in the progress of the disease and influence the deterioration of lung function [31–33]. Compliance to treatment is most probably another factor of importance. Even if BPI-ANCA heralds an unfavourable host-pathogen interaction with *PsA*, there are always a multitude of factors affecting outcome, deflating the statistical value of the predication based on the test. The number of patients in this study is unfortunately too low to do a multivariant analysis where all known factors could be compared, which is a shortcoming of this study.

In this cohort of 46 patients, IgA-BPI-ANCA, lung function, and *PsA* colonization have been followed over time, and we see patients who at the beginning of the study belong to one colonization group, but who over the time acquire a different status. The timely relation of when a patient acquires *PsA* and possibly thereafter IgA-BPI-ANCA would be interesting to study. It has been shown possible to influence the BPI-ANCA level by lung transplantation and sinus surgery [18] and the possible BPI-ANCA change after PsA treatment would be another issue to study.

*PsA* serologies are used to detect early infection in order to allow eradication of the bacteria before it becomes a chronic habitant in the lungs. Titres are also used to classify patients as chronic or not [24, 34, 35]. The relation between BPI-ANCA and different *PsA* serologies has so far not been studied. It is not known if BPI-ANCA yields the same set of information as available *PsA* serology tests or not. The fact that most ANCA-positive patients in the Leeds IV group remained free of PsA hints that BPI-ANCA is not a reliable tool to find early colonization. The finding that some patients in Leeds group I remained ANCA negative over a decade also suggests that BPI-ANCA is something else than *PsA* serology test. The relationship between BPI-ANCA and *PsA* serologies needs to be more thoroughly addressed. 

## 5. Conclusion

The results of this study show that BPI-ANCA, a single serological test at one occasion, is a prognostic biomarker for the long-term outcome among adult CF patients and suggest that it has better ability to predict prognosis than a classification based on multiple sputum cultures. However, it is not clear if BPI-ANCA (or Leeds criteria) adds additional prognostic information regarding development of end-stage lung disease when the degree of lung function impairment is known. Larger studies are needed to establish the usefulness of BPI-ANCA in different clinical situations.

## Figures and Tables

**Table 1:** Characteristics of the adult CF cohort at the time of BPI-ANCA sampling (1995–1998).

Number of patients: (*n*)	
Total	46
Males	26
Females	20
Age: (years)	
Mean	26.2
Range	18.4–44.6
CFTR mutation: (*n*)	
ΔF508del/ΔF508del	24
Others	22
FEV1.0 % predicted: (*n*)	
>80%	16
50–80%	17
<50%	13
IgA BPI-ANCA:	
Negative (≤67 U)	
(*n*)	17
(mean age, years)	27.5
Positive (>67–200 U)	
(*n*)	18
(mean age, years)	26.3
High (>200 U)	
(*n*)	11
(mean age, years)	24.6
Leeds classification of *PsA* colonization: (*n*)	
I (chronic)	24
II (intermittent)	2
III (free of *PsA*)	8
IV (never *PsA*)	12
Diabetes mellitus: (*n*)	
Yes	5
No	34
NA	1

**Table 2:** *PsA* colonization, BPI-ANCA, and outcome. Adult CF-patients divided into groups based on Leeds classification and IgA BPI-ANCA levels at baseline and subsequent endpoints during followup.

Leeds classification	Leeds I + II			Leeds III			Leeds IV		
BPI-ANCA level	BPI−	BPI+	BPI++	BPI−	BPI+	BPI++	BPI−	BPI+	BPI++
Inclusion (*n*)	5	12	9	6**	1	1	6	5*	1
5 years % with endpoint	0	16.7%	33.3%	16.7%	0	0	0	20%	0
(*n* with endpoint)		(2)	(3)	(1)				(1*)	
10 years % with endpoint	0	33.3%	77.8%	33.3%	0	100%	0	20%	0
(*n* with endpoint)		(4)	(7)	(2**)		(1)		(1*)	
Last followup, % with endpoint	20%	41.7%	88.9%	33.3%	0	100%	0	40%	0
(*n* with endpoint)	(1)	(5)	(8)	(2**)		(1)		(2*)	

*Patient with colon cancer.

**Patient with *Burkholderia cepacia*.

**Table 3:** Lung function, BPI-ANCA, and outcome. Patients divided into groups based lung function and IgA BPI-ANCA levels at baseline, and subsequent end-points during followup.

Lung function	FEV1 pred > 80%			FEV1 pred 50–80%			FEV1 pred < 50%		
BPI-ANCA group	BPI−	BPI+	BPI++	BPI−	BPI+	BPI++	BPI−	BPI+	BPI++
Inclusion (*n*)	11	5	0	5**	7*	5	1	6	6
5 years % with endpoint	0	0	—	20%	14.3%	20%	0	33%	33%
(*n* with endpoint)				(1)	(1*)	(1)		(2)	(2)
10 years % with endpoint	0	0	—	40%	28.6%	40%	0	50%	100%
(*n* with endpoint)				(2**)	(2*)	(2)		(3)	(6)
Last followup, % with endpoint	0	0	—	40%	42.8%	60%	100%	66.7%	100%
(*n* with endpoint)				(2**)	(3*)	(3)	(1)	(4)	(6)

*Patient with colon cancer.

**Patient with *Burkholderia cepacia*.
